# Destruction of Chitosan and Its Complexes with Cobalt(II) and Copper(II) Tetrasulphophthalocyanines

**DOI:** 10.3390/polym13162781

**Published:** 2021-08-19

**Authors:** Natalia Sh. Lebedeva, Elena S. Yurina, Sabir S. Guseinov, Yury A. Gubarev, Anatoly I. V’yugin

**Affiliations:** G.A. Krestov Institute of Solution Chemistry of the Russian Academy of Sciences, 153045 Ivanovo, Russia; nsl@isc-ras.ru (N.S.L.); yurina_elena77@mail.ru (E.S.Y.); ssg@isc-ras.ru (S.S.G.); aiv@isc-ras.ru (A.I.V.)

**Keywords:** chitosan, phthalocyanines, destruction, complexes, TG/DTG, c-DTA

## Abstract

Chitosan is a naturally occurring polysaccharide derived from chitin with a wide range of uses. Phthalocyanines are macroheterocyclic compounds that have a number of useful properties such as coloring and catalytic and antioxidant activity. Phthalocyanines are able to immobilize on chitosan, forming complexes with new useful properties. In this work, we evaluated the ability of phthalocyanines to increase the thermal stability of chitosan. Chitosan (CS) forms complexes with copper(II)-(CuPc) and cobalt(II)-(CoPc) tetrasulphophthalocyanines. The processes of destruction of chitosan (CS) and its complexes with sulphophthalocyanines CuPc and CoPc in oxidizing and inert atmospheres have been studied. It was established that, regardless of the atmosphere composition, the first chemical reactions taking place in the studied systems are elimination reactions. The latter ones in the case of chitosan and complex CS-CuPc lead to the formation of spatially crosslinked polymer structures, and it causes the release of CuPc from the polymer complex. It was found that in the case of CS-CoPc elimination reactions did not lead to the formation of crosslinked polymer structures but caused the destruction of the pyranose rings with a partial release of CoPc. Metallophthalocyanines showed antioxidant properties in the composition of complexes with chitosan, increasing the temperature of the beginning of glycosidic bond cleavage reaction by 30–35 °C in comparison with the similar characteristics for chitosan.

## 1. Introduction

Phthalocyanines are aromatic macroheterocyclic compounds capable of forming internal salts with almost all metals of the Periodic system. In this case, the aromatic phthalocyanine macrocycle is an equatorial ligand. Stable intracomplex of phthalocyanines salts with metal ions arises due to the formation of four equivalent σ -bonds N ->M (i.e., when filling vacant s-, p_x_-, p_y_-, (n-1)d_x_^2^_y_^2^-, nd_x_^2^_y_^2^- orbitals of a metal ion with σ-electrons of nitrogen atoms of the phthalocyanine reaction center). Among the factors determining the stability of complexes of phthalocyanines with metal ions, the geometric correspondence of the covalent radius of the metal cation and the diameter of the reaction cavity of the macro ring are of great importance. They determine the efficiency of the formation of σ- and π-coordination bonds (metal-phthalocyanine). For example, the diameter of the phthalocyanine reaction center is about 2.8 Å [1] and it almost perfectly corresponds to the covalent radius of copper and cobalt atoms, which are 2.6 and 2.5 Å, respectively. Structural-energy correspondence leads to the formation of metal complexes of phthalocyanines (MPc) that are resistant to electrolytic dissociation and the action of strong inorganic acids. In the presence of a residual positive charge on the metal, in case of its coordination unsaturation, the possibility of forming axial coordination of electron-donating ligands appears. The coordination donor—acceptor interaction of MPc with molecular ligands significantly changes the electrochemical and spectral properties of MPc, and this phenomenon finds application in creating sensors and analyzers [2]. The aromatic π-system of MPc determines their ability to absorb light in the visible part of the spectrum. According to some evaluations, phthalocyanines are the second most important class of dyes, and copper phthalocyanine is the best-selling dye [3]. Based on MPc, pigments were developed for automotive paints and printing inks, as well as light blue/blue dyes for textiles and paper, for inkjet printing, electrophotography. MPc during photo-irradiation are capable of generating singlet oxygen and other active forms of oxygen, and this feature of them is used in the treatment of cancer by means of photodynamic therapy [4]. The ability of the compounds of the porphyrin and phthalocyanine class to catalyze the reactions of epoxidation [5,6,7], oxidation [8,9,10,11], including reactions in which molecular oxygen acts as an oxidizing agent [10,12], is widely known. It is found that Co(II)phthalocyanine can transfer oxygen from various oxygen donors to alkanes, alkenes, phenols and thiols in the several studies [10,13,14,15]. The basis of catalysis reactions is a decrease in the activation energy of the process due to the formation of intermediate compounds and/or changes in the oxidation state of the metal phthalocyanine. MPc are rightfully among the most promising organic compounds in modern chemistry.

Recently, publications began to appear in the scientific literature that have reported on the diametrically opposite properties of MPc—their antioxidant ability [16,17,18,19,20,21,22]. Evaluation of the antioxidant activity of MPc is usually carried out with several test systems: (1) upon inactivation of the radical 2,2-diphenyl-1-picrylhydrazyl [18,23] (DPPH), which is due to the presence of an odd electron has an intense absorption band in the UV-Vis spectrum at 517 nm, and when an electron or hydrogen atom is accepted from an antioxidant it goes into a nonradical form and changes its color from violet to yellow; (2) by analysis of the absorption of superoxide radicals O_2_^‒^. In this method, the amount of inactivated radical superoxide anions is estimated by the antioxidant inhibition of the blue dye Nitroblue tetrazolium (NBT) formed in the reaction between the superoxide anion and the yellow dye (NBT^2+^) [24]; (3) according to the evaluation of the regenerative ability of antioxidants. In this method, reducing activity was based on the reduction of Fe^3+^/ferricyanide complex to the ferrous form in the tested samples with reductants (antioxidants). The Fe^2+^ was then monitored by measuring the formation of Perl’s Prussian blue at 700 nm. Judging by numerous data [16,17,18,19,20,21,22], the antioxidant ability of MPc is usually higher than the most well-known antioxidants (such as ascorbic acid and Trolox), including butylhydroxytoluene. It should be noted that the mechanism of manifestation of antioxidant activity by MPc is not completely understood, and the interpretation of the observed phenomenon by different authors varies significantly. Nevertheless, this is an extremely interesting phenomenon both scientifically and practically, since the antioxidant ability of MPc, along with its coloring and catalytic properties, can become the basis for developing new polymer compositions with an increased oxidation resistance, while retaining mechanical strength and color of natural and synthetic polymers.

Chitosan is one of the natural polymers that are currently being widely studied. Chitosan is a biodegradable polymer, and therefore it is very popular in terms of green chemistry [25]. New materials for photodynamic therapy, photocatalytic removal of pollutants, and water and air purification are being developed on the basis of chitosan and phthalocyanines [26,27,28]. Metallophthalocyanines can exhibit antioxidant activity in the thermal oxidation of chitosan. Perhaps they will change the mechanism of thermal oxidation of polysaccharides and lead to the generation of various pyrolysis products that may have commercial value.

Therefore, the aim of this work was to study the effect of metal complexes of sulphosubstituted phthalocyanines on the process of thermal oxidative destruction of chitosan on air and compare the results obtained with destruction in an inert atmosphere. At the first stage of the work, it is necessary to obtain polymer complexes of chitosan with phthalocyanines. Due to the fact that the studied chitosan and phthalocyanines are water-soluble, the complexes were obtained by mixing solutions of chitosan and phthalocyanine and subsequent drying on air. The thermochemical study of chitosan was carried out using TG/DTG, c-DTA, and mass spectrometric analysis. The obtained new fundamental knowledge can be useful for future applications using chitosan-phthalocyanine complexes.

## 2. Materials and Methods

### 2.1. Materials

**Scheme 1:**
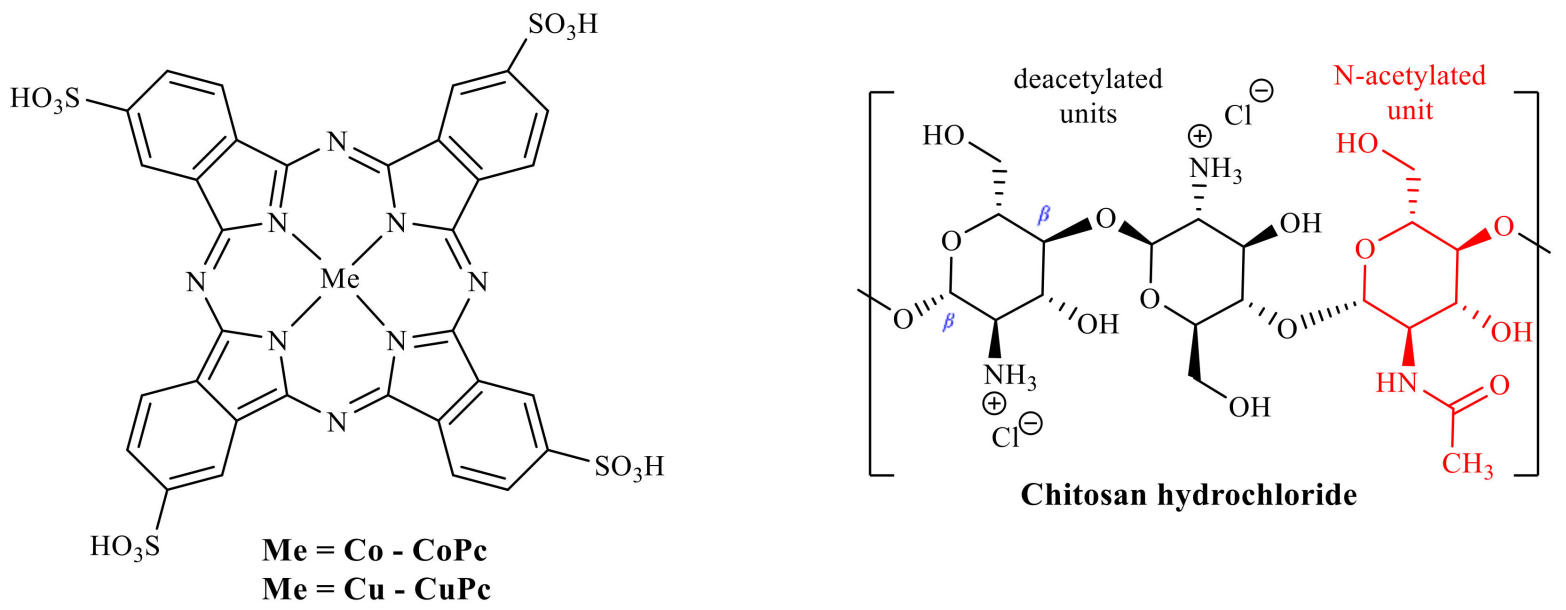
Structural formulas of metal complexes of tetrasulphophthalocyanines and chitosan. Chitosan composed of randomly distributed β-(1→4)-linked D-glucosamine (deacetylated unit) and N-acetyl-D-glucosamine (acetylated unit).

Herein we used chitosan hydrochloride (Scheme 1) from crab shells (Bioprogress, Russia) with an average viscosity molecular weight of 18.8 kDa, which was determined using the Mark–Kuhn–Hauwink equation:

(1) $$\left[\eta \right]\;=K\cdot M^{\alpha },$$

where [η] is the intrinsic viscosity of a polymer chain with a molecular weight M, α and K are the constants magnitude which depend on the nature of the polymer and solvent, and on the temperature. For chitosan in the acetate buffer at 25 °C the constants were: K = 1.464 × 10^−4^; α = 0.885 [29]. The degree of deacetylation of chitosan was determined by the method of ^1^H NMR spectroscopy described in [30]. ^1^ H NMR spectra were recorded with an Avance III (Bruker) NMR spectrometer (500.17 MHz) using a 2% DCl solution in D_2_O as solvent. The degree of deacetylation of the sample used in the work was 94%. pH of chitosan solution was 6.5.

Copper(II)tetrasulphophthalocyanine and cobalt(II)tetrasulphophthalocyanine (Scheme 1) were synthesized and purified according to the methods in [31]. The degree of purity of the tetrasulphophthalocyanines used in the work was not less than 98%. The studies were carried out in double distilled, deionized water, electrical conductivity at 25 °C, 0.5–1.0 μs·cm^−1^.

Crystalline samples of polymer complexes of chitosan with metallophthalocyanines were obtained by evaporating solutions with a concentration of sulphophthalocyanines 6.4 × 10^−5^ M and 0.02 mass% of chitosan.

DMF with a purity greater than 99%, ethanol 98% and chloroform with a purity of 99.5% were used as received from Sigma Aldrich without further purification.

### 2.2. Spectral Investigation

UV-Vis spectra of solutions were registered using AvaSpec-2048 spectrophotometer (Avantes BV, Apeldoorn, Netherlands), in 1 cm quartz cuvettes at 25 °C. AvaLight-DHS was used as light source.

### 2.3. TG/DTG

For thermogravimetric analysis a thermo-microbalance TG 209 F1 (Netzsch Geratebau GmbH, Germany) was used. Powdered samples (4–7 mg) were placed in platinum crucibles and heated at a rate of 10 °C·min^−1^ in a dynamic atmosphere air with a gas flow rate of 30 mL·min^−1^ from room temperature to 600–700 °C. The accuracy of measuring the mass of the sample amounted 1 × 10^−7^ g. The supplied software allows the semi-quantitative determination of thermal effects using the c-DTA curve. c-DTA is the calculated differential thermal analysis (DTA) curve. The c-DTA signal is evaluated as the difference between the temperature-time curve with the test sample and the temperature-time curve without the sample (baseline with an empty crucible). Despite the low sensitivity of the c-DTA signal, compared to the DSC signal, it can be used to identify thermal effects in the process of thermal and thermooxidative decomposition of the samples [32].

### 2.4. Mass Spectrometric Analysis

Mass spectrometric analysis was carried out using a STA 409 CD thermoanalytical unit (Netzsch Gerätebau GmbH, Germany) equipped with a mass spectrometer with a QMG 422 skimmer system (In Process Instruments, Germany). This unit provided thermogravimetric and DSC measurements with simultaneous recording of mass spectra of the thermal decomposition products. The samples were heated at atmospheric pressure in a stream of dry ultrapure argon (70 mL·min^−1^).

## 3. Results and Discussion

### 3.1. Preparation of MPc-Chitosan Complexes

**Figure 1:**
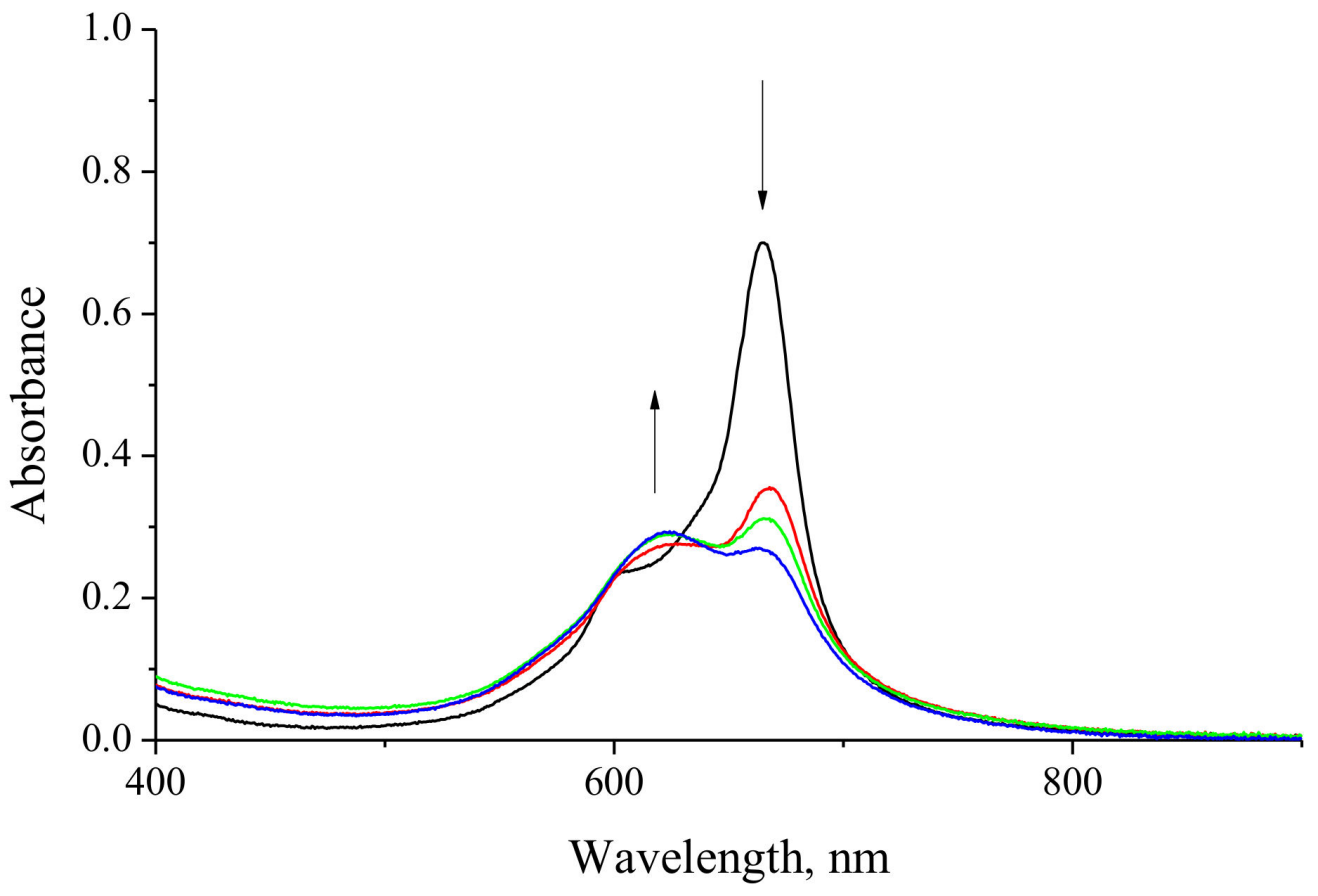
UV-Vis spectra of CoPc (2 × 10^−5^ M, black line) with addition of chitosan 0.01 mass% (red line), 0.015 mass% (green line) and 0.02 mass% (blue line).

There are various approaches to obtaining chitosan complexes with phthalocyanines. For example, in [25], the ability of the mentioned polysaccharide consisting of N-acetyl D-amine and D-glucosamine units to bind d-metal ions due to the formation of donor-acceptor bonds of the M←NH_2_ group of chitosan, with further synthesis of MPc In Situ was used. For this, phthalonitrile was added to the resulting complex, and the reaction was carried out in a deep eutectic solvent (DES) at 150 °C for 1 h. The approach is rather interesting, but we failed to achieve 100% monomerization of MPc within this complex. Possible reasons are either a change in the conformation of the natural polymer in the DES medium (choline chloride: urea in a molar ratio of 2:1), or weak retention and migration of CuPc in the composition of the polymer. The only type of bond between synthesized copper(II) phthalocyanine and chitosan is a very weak donor—acceptor bond of the Cu←NH_2_ of chitosan group, if any. Cu^2+^ (3d^9^), in addition to the formation of σ-bonds with the phthalocyanine macrocycle, can form inverse native π-bonds of metal ion→phthalocyanine, and this leads to an extremely low coordination ability. In addition, the authors of [25] did not evaluate possible changes in the chemical composition of chitosan itself upon heating. Therefore, in this work, we used a differ approach, successfully proven itself earlier [33,34], without using high temperatures and based on the formation of complexes between anionic sulphoderivatives of MPc and a cationic polysaccharide. Briefly, the procedure was as follows: a solution of chitosan hydrochloride was dissolved in deionized water and the required amount of metallosulphophthalocyanine was added with stirring and keeping to establish possible aggregation equilibria. Typical spectral changes of the binding of MPc with chitosan are presented in Figure 1.

Before the addition of chitosan, CoPc (2 × 10^−5^ M) has an electronic absorption spectrum typical of MPc in the monomeric state (namely the Q-band) is clearly resolved with a maximum at 667 nm and vibrational satellite at 599 nm (Figure 1). CuPc (2 × 10^−5^ M) in the initial solution is partially dimerized, since in addition to the absorption of the monomer (λ = 657 nm), absorption by dimeric structures (632 nm) is also recorded. The addition of chitosan to the phthalocyanine solution leads to an increase in the proportion of dimeric structures in both cases. The spectral changes (Figure 1) are consistent with previously obtained data for the indicated systems [33,34] and correspond to the established interaction mechanism according to which CuPc binds to chitosan in the dimeric state due to electrostatic and H-bonding between the peripheral SO_3_^−^, CuPc, and NH^+^ groups and chitosan groups. Judging by the previously obtained IR spectra [33] for CoPc, in addition to electrostatic and H-bonding between peripheral CoPc substituents and chitosan groups, the formation of donor—acceptor bonds of the Co←NH_2_ group of chitosan was found. It should be noted that the advantages of the applied method are its simplicity, economy, easy isolation of the polymer complex of chitosan-MPc in solid form, and the possibility of introducing the required amount of MPc into chitosan. Thus, complexes of phthalocyanines with chitosan were obtained in an aqueous medium, and they were isolated in the solid state.

### 3.2. Thermochemical Study

**Figure 2:**
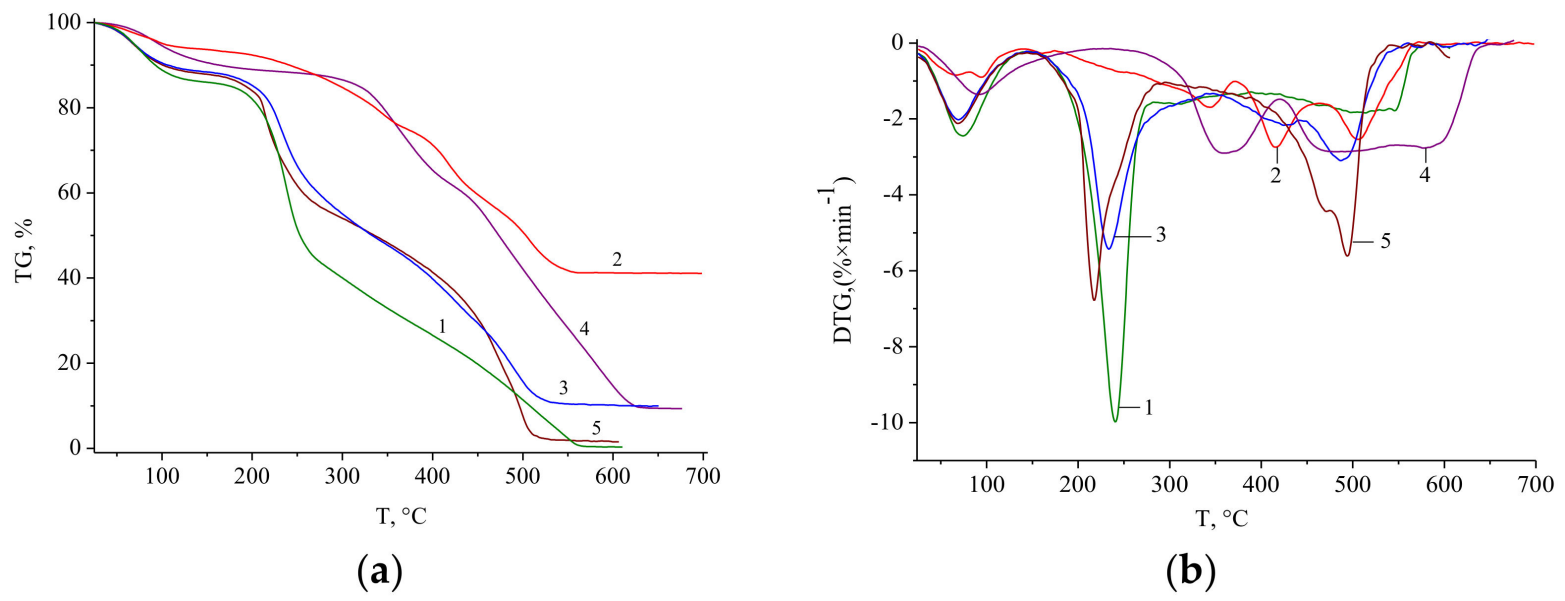
TG (**a**) and DTG (**b**) curves obtained for thermo-oxidative degradation of pure compounds-CS (line 1), CoPc (line 2), CuPc (line 4) and their complexes-CS-CoPc (line 3) and CS-CuPc (line 5) at a heating rate of 10 °C·min^−1^ in air.

In Figure 2 the thermograms of the mass change (TG) of samples CS, CoPc, CS-CoPc, CuPc, and CS-CuPc in the temperature range from room temperature to 600–700 °C in air are shown. The mass loss of all samples upon heating occurs in four stages and begins with the evaporation of adsorbed water in the temperature range from room temperature to 135 °C. To interpret the differences in the behavior of the samples under heating, the main parameters of thermal oxidative decomposition (Ton, T_max_, T_end_, and Δm) were determined from the TG/DTG curves and are presented in Table 1.

As can be seen from Table 1, the change in the mass of the polymer complexes CSCoPc (11.5%) and CSCuPc (11.9%) at the initial stage (initial mass loss) due to the water removal is less than for chitosan (13.6%). This fact indicates that the CS-CoPc and CS-CuPc complexes contain fewer free amino groups available for the formation of hydrogen bonds with water molecules, since some of the amino groups of chitosan participate in the complexation process with CoPc and CuPc, and this fact further confirms the proposed model of interactions [34].

**Figure 3:**
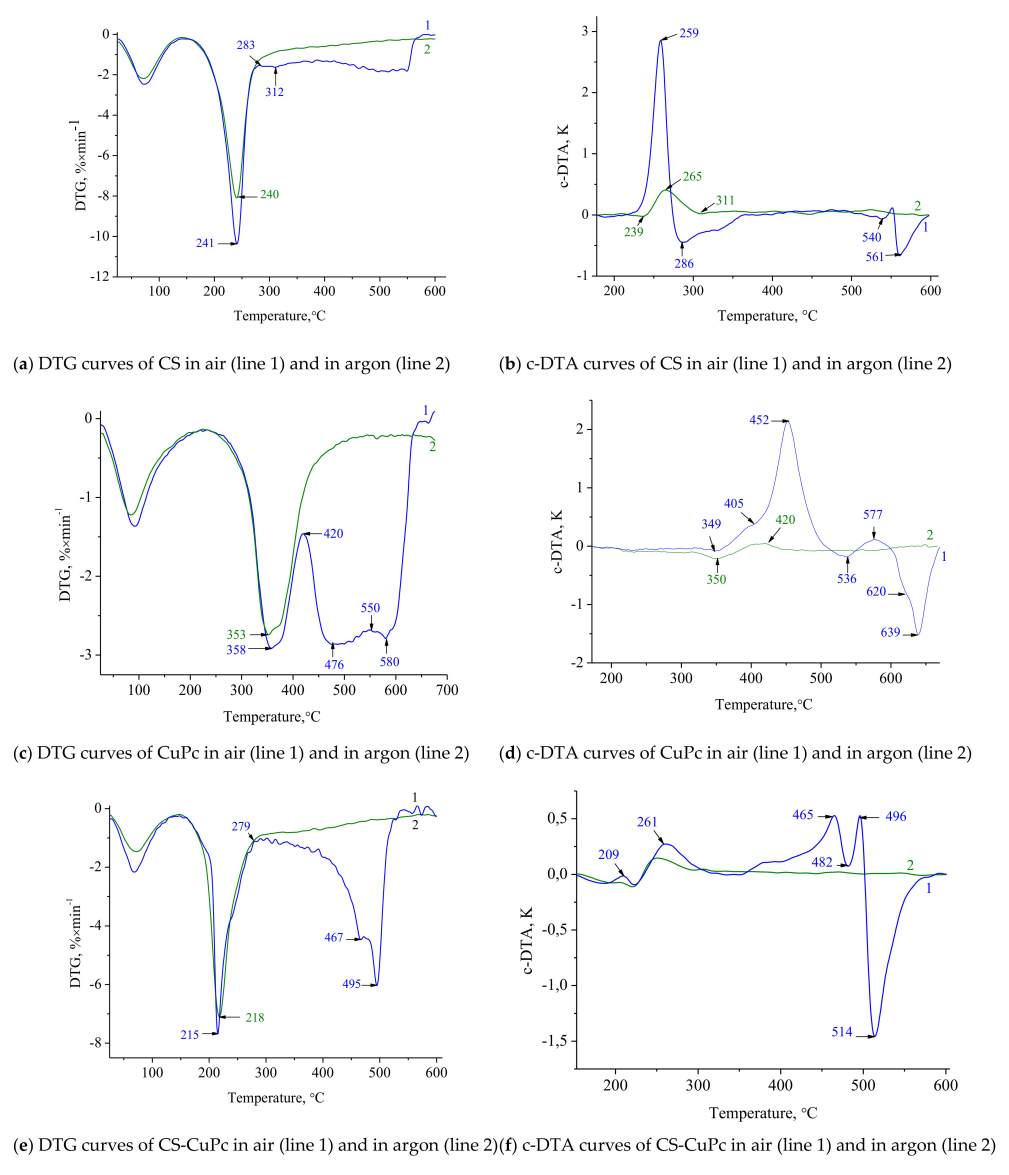

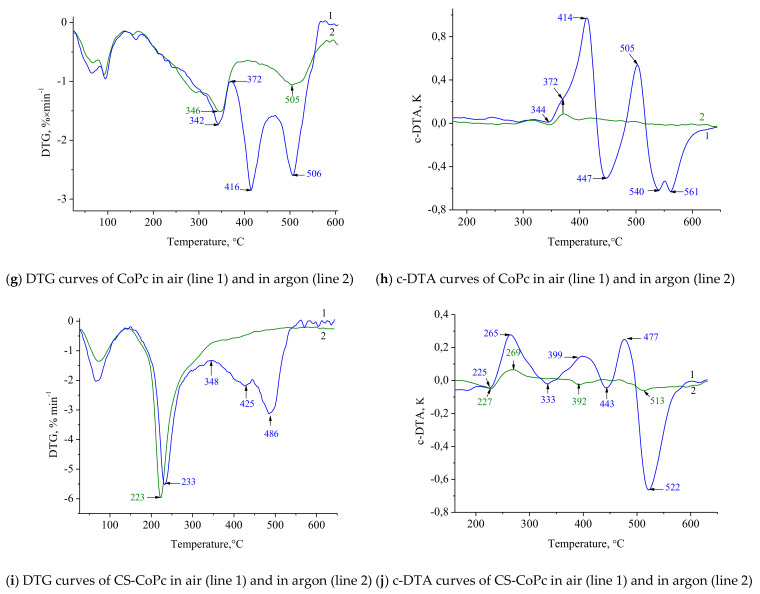
DTG and c-DTA curves of individual CS, CoPc, CuPc and their complexes CS-CoPc and CS-CuPc.

The next stage after dehydration is the beginning of the process of thermooxidative degradation of the polymer and its complexes with MPc. The interpretation of the chemistry of the thermal oxidation process, in contrast to pyrolysis, is more complicated, since the use of an atmosphere of air excludes the possibility of recording mass spectra of gaseous products, and the analysis of the IR spectra of samples heated to certain temperatures is complicated due to the ambiguity of spectral changes. Therefore, it makes sense to establish the general and distinctive features of the destruction of CS, CoPc, CS-CoPc, CuPc and CS-CuPc in air obtained in this work and the thermal decomposition of the same samples in argon obtained in the previous work [35] (Figure 3).

As can be seen from Figure 3, the course of the DTG curves of the studied processes at the initial stages coincides. Within the error, the change in mass measured from the temperature of the start of thermooxidative/thermal decomposition of the samples (according to the DTG curve) and the temperature (T_2_) to which the DTG curves in argon and air change symbatically coincide (Figure 3).

The data obtained indicate that the chemistry of the process in argon and the air atmosphere up to temperatures T_2_ is identical. Therefore, thermolysis occurs in the analyzed temperature range and air oxygen is not involved. Let us analyze the results obtained according to the systems.

#### 3.2.1. Pyrolysis and Thermo-Oxidative Destruction of Chitosan

Since this system is fairly well studied, we will dwell on the key points. Judging by the TG curves (Figure 1), the destruction of chitosan in air begins when reaching 215 °C, this temperature being slightly higher than in argon at 212 °C [34]. It should be noted that this stage is superimposed on the previous stage (dehydration) and this phenomenon will introduce an error in determining the temperature of destruction (and, possibly, will overestimate the real value). Indeed, as shown by a previous mass spectral study of chitosan pyrolysis products in argon [35], when reaching 152–159 °C, a weak CO_2_ emission is recorded in the mass spectra due to random breaking of glycosidic bonds, and it leads to the formation of radical products that recombine with each other [36,37]. Upon reaching 172–178 °C, the main emission of gaseous pyrolysis products is conditioned by the release of ammonia, water, acetic acid fragments, hydrogen chloride, and this condition corresponds to chemical deamination, intermolecular dehydration [35], deacetylation, and salt destruction. To a much lesser extent, breaking of glycosidic bonds and destruction of pyranose rings are detected. The latter two reactions proceed most intensively at higher temperatures (220 °C). In addition to the gaseous products listed above, cross-linked polymer structures appear, the formation of which is accompanied by an exo-effect on the c-DTA curves (Figure 3). It is in the analysis of c-DTA curves those differences are observed in the destruction of chitosan in an inert and oxidizing atmosphere. The thermal effect of the formation of crosslinked polymer structures is much greater in the air atmosphere compared with an inert atmosphere. Moreover, it begins earlier and reaches a maximum at 259 °C, while the maximum heat release in argon falls at 265 °C (Figure 3). It is likely that in an oxidizing atmosphere a greater number of glycosidic C—O—C bonds undergo degradation. This assumption is supported by a slightly larger mass loss in the analyzed temperature range in air compared with argon (Table 2).

#### 3.2.2. Pyrolysis and Thermal Oxidative Destruction of MPc

Both in an air and in an inert atmosphere, the destruction of metal complexes of sulphosubstituted phthalocyanines begins with the stage of cleavage of peripheral substituents, followed by the stage of the macro ring breaking, at temperatures of 350 °C and above the UV-Vis spectrum of MPc is not reproduced. In the air atmosphere the hydrocarbons obtained in the previous stages are oxidized to the formation of the corresponding higher oxides, and this process is accompanied by two exo-effects on the c-DTA curves with maxima of 414, 505 °C and 452, 577 °C for CoPc, and CuPc, respectively.

#### 3.2.3. Pyrolysis and Thermal Oxidative Destruction of CS-CuPc

**Figure 4:**
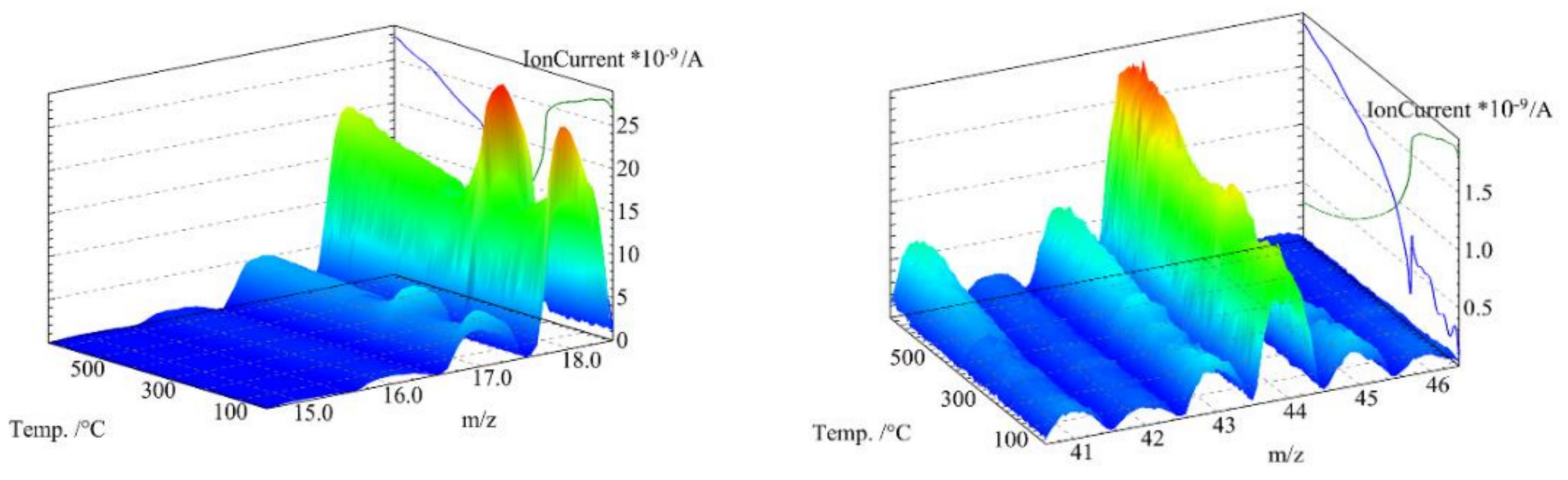
Mass spectra of gases evolved during the thermal treatment of CS-CuPc.

Judging by the TG curves (Figure 1), the destruction of the CS-CuPc polymer complex in an oxidizing atmosphere begins at 204 °C, and in argon at 200 °C. The destruction processes of CS-CuPc in air and in argon coincide up to a temperature of 290 °C. As shown previously [35], in contrast to chitosan, starting from a temperature of 164 °C, the first gaseous products of CS-CuPc degradation detected are ammonia, fragments of acetic acid (m/z = 15, 43) (Figure 4) and acetic anhydride (m/z = 42) (i.e., the destruction process of CS-CuPc proceeds from the stage of deamination, dehydration, and deacetylation). Upon reaching 188 °C, reaction products of randomly breaking glycosidic bonds are recorded in the mass spectra [35], and this temperature is approximately 30 °C higher than at the start of the temperature of destruction of glycosidic bonds of pure chitosan. Starting from 195 °C, gaseous hydrogen chloride is removed. The exo-effect on the c-DTA CS-CuPc curve is slightly larger in the oxygen atmosphere, and its maximum is shifted to the high temperature region (Figure 3) in contrast to chitosan. As was shown above, for chitosan the replacement of an inert atmosphere by an air one leads to a multiple increase in exo-effect with a maximum shifted to lower temperatures regions (Figure 3).

**Figure 5:**
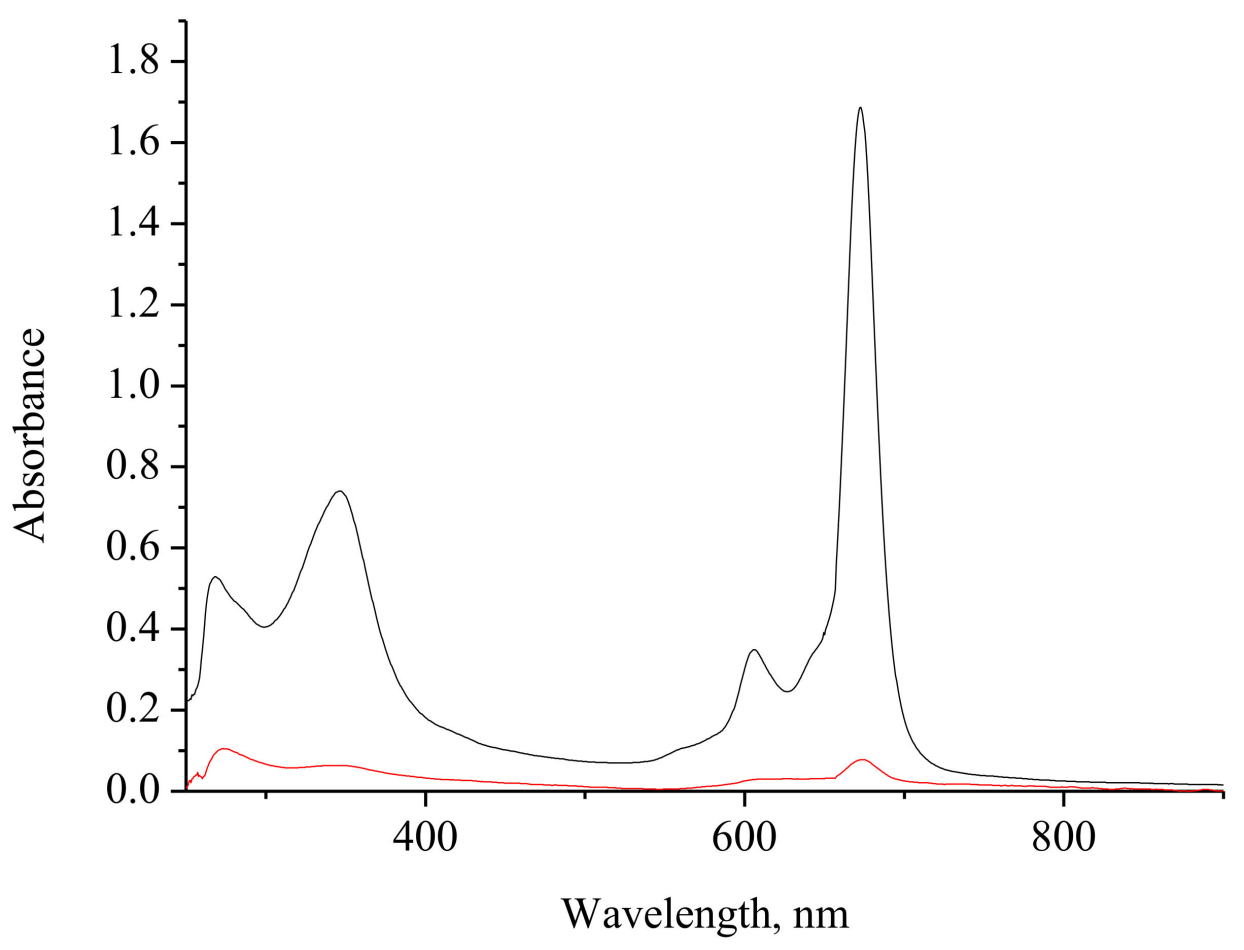
UV-Vis spectra of CuPc in DMF obtained by extraction from the initial CS-CuPc (red line) and heated to 225 °C CS-CuPc (black line) with DMF.

We also conducted a qualitative study of the solubility of the initial polymer CS-CuPc complex and the one heated to a temperature of 225 °C in water, in acidified water, in chloroform, in ethyl alcohol and in DMF. It turned out that the blue-green initial polymer complex is highly soluble in water and insoluble in organic solvents. In DMF a partial transition of CuPc to DMF was observed, and this phenomenon indicates the high strength of the CS-CuPc complex (Figure 5). Black CS-CuPc heated to 225 °C is insoluble in aqueous and organic solvents. However, CuPc is easily extracted with DMF (Figure 5).

It is likely that deamination and dehydration reactions lead to the formation of crosslinked polymer structures, while eliminating the possibility of keeping the primary electrostatic and H-bonds between the sulphogroups of CuPc and the side groups of the polymer. The incorporation of the CuPc phthalocyanine macrocycle into crosslinked polymer structures is also impossible, since destruction of peripheral substituents begins at higher temperatures (at 269 °C) [35]. In this regard, the CS-CuPc complex is actually destroyed and, in fact, the sample heated to 225 °C is a mixture of copper(II)tetrasulphophthalocyanine with chitosan destruction products, and an easy extraction of CuPc with dimethylformamide is provided.

Thus, to summarize the observations, one can say that the complexation of chitosan with CuPc promotes the processes of deamination, deacetylation and dehydration, probably due to the facilitation of the removal of gaseous products, since large dimeric structures (CuPc)_2_ in the polymer will disorder the packaging of the polymer chains, and disrupt the network of hydrogen bonds between the aminogroups of neighboring macromolecules. The complexation of chitosan with CuPc prevents the occurrence of radical processes associated with the breaking of the glycosidic bond. Thus, CuPc in the composition of the polymer complex of CS-CuPc exhibits antioxidant properties.

#### 3.2.4. Pyrolysis and Thermal Oxidative Destruction of CS-CoPc

Judging by the TG curves (Figure 1), the destruction of the CS-CoPc polymer complex in an oxidizing atmosphere begins at 210 °C, and in argon at 204 °C. The course of the TG and DTG curves of CS-CoPc in air and in argon coincides to a temperature of 342 °C (Figure 3 and Table 2). It indicates that the chemistry of the process coincides. Judging by the previously obtained data in argon [35], the process of random breaking of glycosidic bonds in the polymer complex begins at a temperature of 35 °C higher than in the case of chitosan and 5 °C higher than in the case of CS-CuPc. The deamination and dehydration reaction of CS-CoPc starts at the same temperature as for chitosan (171 °C). Compared to chitosan, CS-CoPc enters the deacetylation reaction at a higher temperature by 5–11 °C. The main differences in the degradation of CS-CoPc compared to CS-CuPc are recorded at higher temperatures, namely, when the temperature reaches 211 °C, and volatile products of the destruction of pyranose rings are recorded in the mass spectra of CS-CoPc. In addition, the c-DTA curve has an unusual appearance, not typical of chitosan and its derivatives, namely, up to a temperature of 350 °C, exo-effects corresponding to the formation of crosslinked polymer structures are not observed (Figure 3).

**Figure 6:**
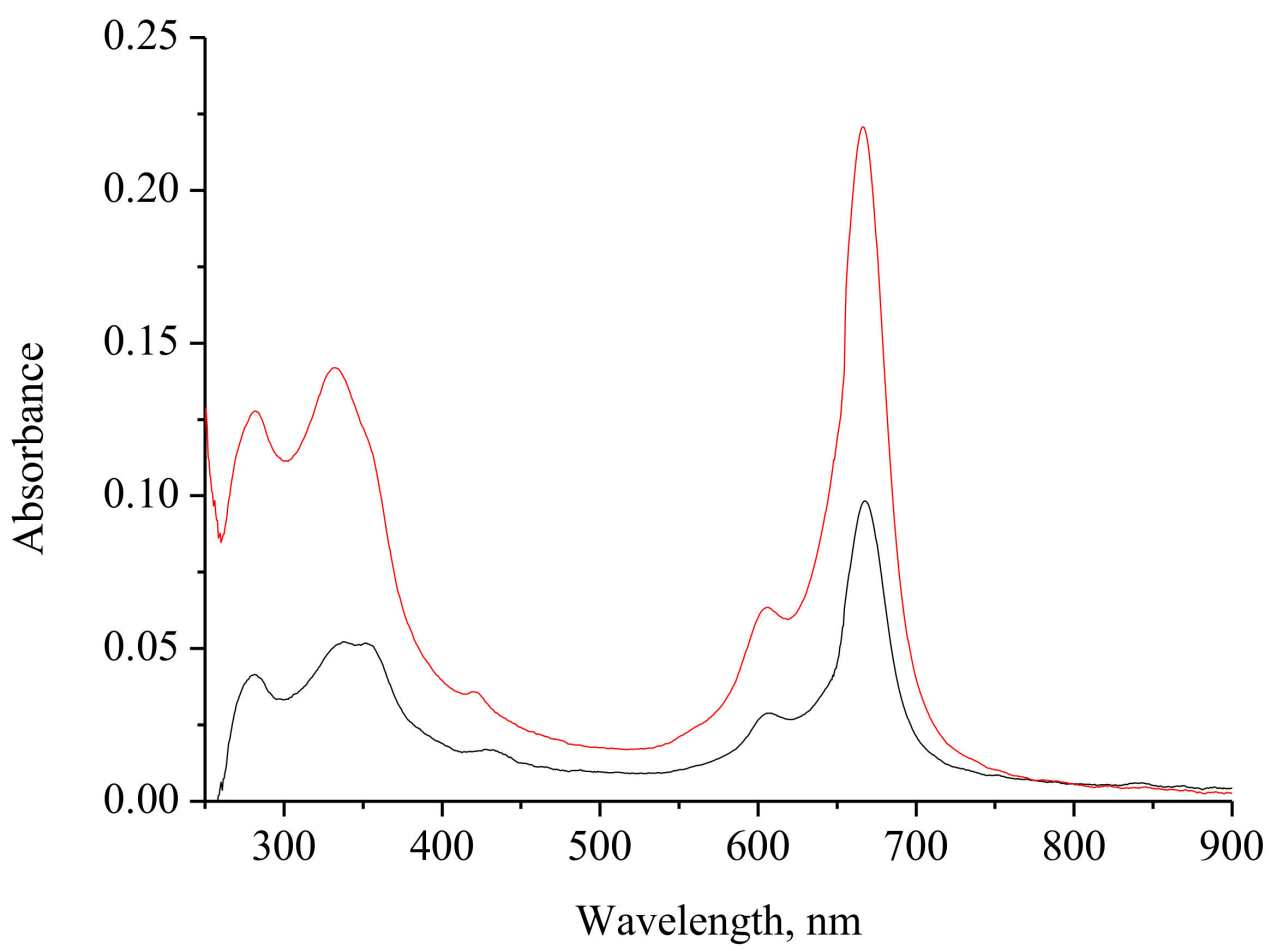
UV-Vis spectra of CoPc in DMF obtained by extraction from the initial CS-CoPc (black line) and heated to 225 °C CS-CoPc (red line) with DMF.

Evaluation of the solubility of the initial CS-CoPc complex and the one heated to a temperature of 225 °C in water, acidified water, chloroform, ethyl alcohol, and DMF showed that the dark blue polymer complex is highly soluble in water and insoluble in organic solvents. As in the case of the initial CS-CuPc complex, CS-CoPc not subject to heating is rather resistant to extraction, and only a partial transition of CoPc to DMF is detected (Figure 6). Heated to 225 °C black CS-CoPc is insoluble in aqueous and organic solvents. Upon prolonged extraction and ultrasonic exposure, CoPc partially turns to a DMF solution (Figure 6).

A lower degree of extraction of CoPc compared to CuPc is probably due to the ability of the Co^2+^ ion in the phthalocyanine to additional coordination on the electron-donating atoms of the polymer (N, O), and the absence of spatially cross-linked polymer structures will not render steric hindrances for the implementation of the indicated donor-acceptor interaction.

Thus, summarizing the data obtained, it can be concluded that the presence of CoPc in the polymer in an oxidizing and inert atmosphere has practically no effect on the temperature at the start of deamination, deacetylation and dehydration of the polymer, but unlike chitosan and CS-CuPc, these elimination reactions do not lead to the formation of crosslinked polymer structures, but cause a collapse of the structure of chitosan and destruction of the pyranose rings. CoPc effectively prevents the occurrence of free radical processes, inactivating randomly generated radicals and thereby breaking the chain.

Upon reaching T_2_ (Table 2), thermal oxidation processes occur in the oxidizing atmosphere, and these processes are accompanied by a mass loss and significant exo-effects (i.e., the combustion process proceeds with the formation of higher oxides).

## 4. Conclusions

The study of the thermochemical behavior of chitosan and its complexes with sulpho-substituted metallophthalocyanines in air was carried out. The results obtained were compared with studies of the pyrolysis of these polymers. It was found that the initial stages of the destruction of chitosan and its complexes with metal sulphophthalocyanines proceed without the participation of atmospheric oxygen.

The destruction of chitosan begins when it reaches 152–159 °C and starts from the stage of breaking the glycosidic bonds. Chemical reactions of deamination, intermolecular dehydration, deacetylation and destruction of the CS-HCl salt proceed when reaching 172–178 °C. The reactions of destruction of glycosidic bonds and pyranose rings proceed with maximum intensity when a temperature of 220 °C is reached. Crosslinked polymer structures are formed during thermal oxidation and pyrolysis at temperatures Tmax = 259 °C in air, Tmax = 265 °C in argon.

It was revealed that the formation of complexes of phthalocyanines with chitosan affects the chemistry of the destruction process. The destruction processes of CS-CuPc in air and in argon are similar up to 290 °C. The destruction of CS-CuPc starts from the stage of deamination, dehydration, and deacetylation. Dimeric structures (CuPc)2 immobilized in the polymer contribute to the elimination reactions due to the better removal of volatile degradation products. The reactions of deamination, intermolecular dehydration, and accidental cleavage of glycosidic bonds lead to the formation of crosslinked polymer structures in the case of chitosan and CS-CuPc. In the latter case it results in the release of CuPc up to T = 225 °C.

CoPc in the CSCoPc complex has a different effect on thermally induced reactions. The chemistry of the processes of destruction of CSCoPc in argon and air are similar up to a temperature of 342 °C. The destruction of CSCoPc, as well as CS, begins from the stage of cleavage of glycosidic bonds. Further, as for CS, the deamination and dehydration reactions of CSCoPc begin when 171 °C is reached. The monomeric and dimeric forms of CoPc immobilized in the polymer do not affect the temperature at which the elimination reactions begin, but at the same time they prevent the formation of crosslinked polymer structures and the release rate of CoPc remains low. It was found that the effect of both metallosulphophthalocyanines on the thermal oxidation of CS is manifested in an increase in the temperature of the onset of polymer-radical reactions by 30–35 °C, associated with the rupture of glycosidic bonds, and this fact can be considered as confirmation of our hypothesis about the antioxidant activity of phthalocyanines.

The information obtained may be in demand in the development of technological processes for the preparation of immobilized catalysts, graphite-like structures and fibers during thermo- and thermo-oxidative action on polysaccharides.

## Figures and Tables

**Table 1:** Parameters of thermogravimetric measurements of dehydration processes and thermal oxidative degradation of CS, CoPc, CS-CoPc, CuPc, and CS-CuPc in air ^a^.

Stage	Temperature	Sample				
		CS	CoPc	CS-CoPc	CuPc	CS-CuPc
Initial mass loss (dehydration)	T_on_ (°C)	52.1	49.9	50.0	62.1	49.9
	T_max_ (°C)	71.6	65.9 94.3	68.7	92.6	68.0
	T_end_ (°C)	101.1	109.4	97.9	134.9	97.0
	mass loss, (%)	13.6	6.1	11.5	11.6	11.9
First stage degradation	T_on_ (°C)	215.2	270.1	210.3	320.7	204.0
	T_max_ (°C)	240.7	341.7	232.7	358.8	215.0
	T_end_ (°C)	254.4	359.6	259.6	393.6	259.3
	mass loss, (%)	43.1	18.2	40.1	26.2	34.1
Second stage degradation	T_on_ (°C)	292.1	399.6	389.7	447.4	430.8
	T_max_ (°C)	311.9	416.3	425.0	475.7	467.1
	T_end_ (°C)	332.2	434.1	440.0	513.0	474.4
	mass loss, (%)	15.5	17.8	18.3	33.9	35.1
Third stage degradation	T_on_ (°C)	454.0	491.4	470.3	564.2	490.3
	T_max_ (°C)	514.5	505.7	485.5	578.5	495.1
	T_end_ (°C)	557.9	537.0	516.5	618.6	509.4
	mass loss, (%)	26.4	16.1	19.8	19.0	17.3

^a^ The onset degradation temperature (Ton) was determined as the temperature corresponding to the crossover of tangents drawn on both sides of the decomposition trace, and the temperature of the maximum mass loss rate was taken as the peak temperature of the degradation (Tmax).

**Table 2:** The mass loss of samples in the temperature range Ton-T2.

Sample	Δm, %		ΔΔm = Δm^Air^ − Δm^Ar^	T_2_, °C
	Ar	Air		
CS	39.1	43.1	4.0	277
CoPc	17.6	17.6	0.0	366
CS-CoPc	39.3	39.7	0.4	342
CuPc	25.3	26.3	1.0	420
CS-CuPc	35.0	32.8	−2.2	290

## Data Availability

Data is contained within the article or Supplementary Material.

## Supplementary Materials

The following are available online at https://www.mdpi.com/article/10.3390/polym13162781/s1, Figure S1. DTG and c-DTA curves of CuPc, CSCoPc.
