# Supplementary material for: Destruction of Chitosan and Its Complexes with Cobalt(II) and Copper(II) Tetrasulphophthalocyanines
Source: Polymers (Basel). 2021 Aug 19;13(16):2781. doi: 10.3390/polym13162781 (PMC8400729; doi:10.3390/polym13162781)
Supplement: Supplementary file 1 [file polymers-13-02781-s001.zip › polymers-1259957-supplementary.pdf]

Supplementary materials: Destruction of chitosan and its complexes with cobalt(II) and copper(II) tetrasulphophthalocyanines

Natalia Sh. Lebedeva, Elena S. Yurina, Sabir S. Guseynov, Yury A. Gubarev and Anatoly I. V'yugin

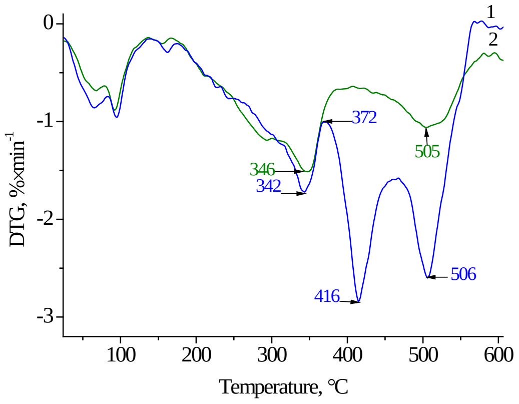

a) DTG curves of CoPc in air (line 1) and in argon (line 2)

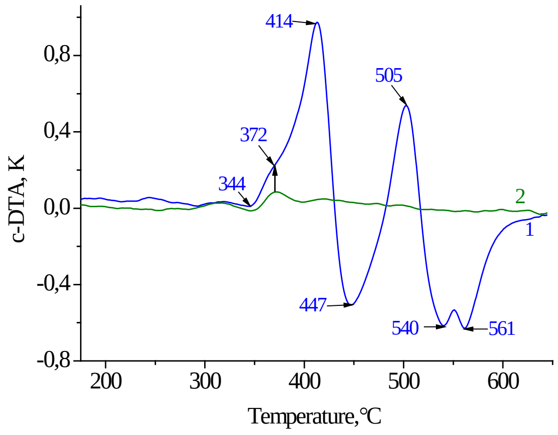

a1) c-DTA curves of CoPc in air (line 1) and in argon (line 2)

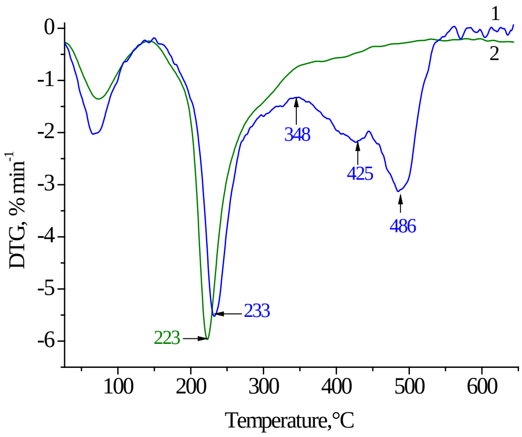

b) DTG curves of CSCoPc in air (line 1) and in argon (line 2)

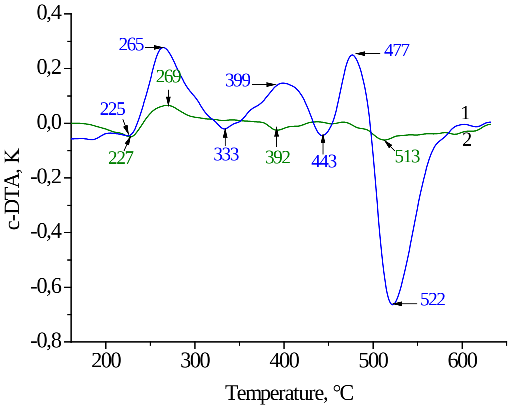

b1) c-DTA curves of CSCoPc in air (line 1) and in argon (line 2)

Figure S1. DTG and c-DTA curves of CuPc, CSCoPc.
